# Effects of a Digital Care Pathway for Multiple Sclerosis: Observational Study

**DOI:** 10.2196/51872

**Published:** 2024-08-07

**Authors:** Märt Vesinurm, Anna Maunula, Päivi Olli, Paul Lillrank, Petra Ijäs, Paulus Torkki, Laura Mäkitie, Sini M Laakso

**Affiliations:** 1 Institute of Healthcare Engineering and Management Department of Industrial Engineering and Management Aalto University School of Science Espoo Finland; 2 Brain Center Department of Neurology Hyvinkää Hospital Hyvinkää Finland; 3 Translational Immunology Research Program University of Helsinki Helsinki Finland; 4 Brain Center Department of Neurology Helsinki University Hospital Helsinki Finland; 5 Department of Clinical Neurosciences University of Helsinki Helsinki Finland; 6 Department of Public Health Faculty of Medicine University of Helsinki Helsinki Finland

**Keywords:** digital care pathway, multiple sclerosis, patient satisfaction, outcome, patient reported outcome measures, resource usage, telemedicine, digital care, outpatient clinic, quality of life, quality of care, communication, caregiver, chronic condition, strategy, long-term, patient engagement, digital health service

## Abstract

**Background:**

Helsinki University Hospital has developed a digital care pathway (DCP) for people with multiple sclerosis (MS) to improve the care quality. DCP was designed for especially newly diagnosed patients to support adaptation to a chronic disease.

**Objective:**

This study investigated the MS DCP user behavior and its impact on patient education-mediated changes in health care use, patient-perceived impact of MS on psychological and physical functional health, and patient satisfaction.

**Methods:**

We collected data from the service launch in March 2020 until the end of 2022 (observation period). The number of users, user logins, and their timing and messages sent were collected. The association of the DCP on health care use was studied in a case-control setting in which patients were allowed to freely select whether they wanted to use the service (DCP group n=63) or not (control group n=112). The number of physical and remote appointments either to a doctor, nurse, or other services were considered in addition to emergency department visits and inpatient days. The follow-up time was 1 year (study period). Furthermore, a subgroup of 36 patients was recruited to fill out surveys on net promoter score (NPS) at 3, 6, and 12 months, and their physical and psychological functional health (Multiple Sclerosis Impact Scale) at 0, 3, 6, and 12 months.

**Results:**

During the observation period, a total of 225 patients had the option to use the service, out of whom 79.1% (178/225) logged into the service. On average, a user of the DCP sent 6.8 messages and logged on 7.4 times, with 72.29% (1182/1635) of logins taking place within 1 year of initiating the service. In case-control cohorts, no statistically significant differences between the groups were found for physical doctors’ appointments, remote doctors’ contacts, physical nurse appointments, remote nurse contacts, emergency department visits, or inpatient days. However, the MS DCP was associated with a 2.05 (SD 0.48) visit increase in other services, within 1 year from diagnosis. In the prospective DCP-cohort, no clinically significant change was observed in the physical functional health between the 0 and 12-month marks, but psychological functional health was improved between 3 and 6 months. Patient satisfaction improved from the NPS index of 21 (favorable) at the 3-month mark to the NPS index of 63 (excellent) at the 12-month mark.

**Conclusions:**

The MS DCP has been used by a majority of the people with MS as a complementary service to regular operations, and we find high satisfaction with the service. Psychological health was enhanced during the use of MS DCP. Our results indicate that DCPs hold great promise for managing chronic conditions such as MS. Future studies should explore the potential of DCPs in different health care settings and patient subgroups.

## Introduction

Multiple sclerosis (MS) is a chronic inflammatory disease of the central nervous system with a continuously rising prevalence [1]. According to the Finnish National MS registry, there are currently more than 12,000 patients with MS in Finland. The mean age of diagnosis is around 30 years for relapsing-remitting multiple sclerosis (RRMS), a subtype of disease that accounts for 90% of patients at diagnosis. MS is the third most common cause for disability pension in Finland [2] and the yearly cost of MS to society was estimated to be roughly €50,000 (US $54,400) per patient in a retrospective cross-sectional questionnaire study [3]. People with multiple sclerosis (pwMS) require long-lasting follow-up by a multidisciplinary team of neurologists, trained nurses, and rehabilitation specialists, to prevent disability accumulation.

Receiving a MS diagnosis is a major event often raising thoughts about a threat to career, social and family life, and independence. Social media is full of information on MS intertwining facts, assumptions, and feelings [4]. After receiving an MS diagnosis, it is common to experience severe reactions of stress [5]. Pharmacological treatment options are various for MS and patients would prefer shared decision-making in choosing the right one for them. Successful shared decision-making is not possible without reliable information [6]. A recently published study investigated barriers and adoption strategies for the early use of high-efficacy therapies in MS [7]. Further, 1 identified adoption strategy is providing patients with sufficient and reliable information, enabling shared decision-making, and improving treatment adherence [7]. From a psychological function perspective, a MS diagnosis has also been shown to cause fatigue, which is an important MS symptom related to underperformance in studies and in work [8].

Digital and telehealth services as an add-on to conventional treatment have shown to be useful in chronic illnesses. A Swedish study found that 63.9% of patients with chronic obstructive pulmonary disease and chronic heart failure found a digital platform and structured telephone support useful [9]. Glycemic control, kidney function, and lipid parameters in diabetics [10] were significantly lower for patients in a telemedicine intervention group compared to the control group, although it should be noted that the intervention and control groups were not completely balanced for socioeconomic factors in this study. An intervention of telehealth with a digital platform showed a significant lowering of systolic blood pressure in a patient group that had poorly controlled hypertension at recruitment [11]. Digital services as an option for a face-to-face model of treatment in osteoarthritis have been shown to have only 25% of the cost of the face-to-face model [12].

eHealth interventions for pwMS have shown varying results. Recent meta-analyses have shown telehealth-based rehabilitation to be an efficient intervention for pwMS [13,14]. Outside of rehabilitation, there are fewer studies on the effects of digital health care programs on pwMS. Further, 1 study found that medication adherence increased slightly from 0.85 to 0.87 in pwMS by a pharmacy and chronic disease treatment program [15]. However, the program did not affect health care use or hospital visits during follow-ups. A web-based self-management platform did not affect the use of health care services of pwMS, and at the end of this study, the control group had a better quality of life [16].

Early support for coping with MS and the change in life is mainly provided by national patient organizations. Online services are easy to use anonymously, and in some countries, these services are of high quality such as in the United Kingdom [17]. A few digital patient portals for pwMS have been created [18,19], but the patient satisfaction and patient-reported outcome measures (PROMs) of these have not been studied. A digital well-being app has been shown to improve well-being in patients with chronic conditions [20], among them pwMS. More established are data on the impact of web-based education programs and services, as shown in MS community members using the Understanding Multiple Sclerosis course [21]. Education on pathogenesis, treatment options, and self-care induced a change in lifestyle and self-management in 44.1% (247/560) of users, and the maintenance of the changed health behavior was measurable in two-thirds of them after 6 months. Similarly, in a randomized controlled trial, a significant reduction in fatigue was observed in pwMS after using a web-based self-management program supported by an email contact [22]. To our knowledge, there is only 1 previous digital health service as an add-on to standard care, aimed to give information about MS, give psychological support, and support lifestyle changes after an MS diagnosis [23]. This was found useful by pwMS.

We hypothesized that a digital health care intervention during the first years after diagnosis of RRMS could show an impact on the care of MS both from the perspective of the patient and the organization. We report on the user behavior on the MS digital care pathway (DCP) and adopt a case-control design to evaluate the health care use 1-year post diagnosis of the MS DCP group (n=63) and control group (n=114). In addition, we report on the PROMs on physical and psychological functional health using the Multiple Sclerosis Impact Scale (MSIS-29) questionnaire and patient satisfaction using net promoter score (NPS) of a separately recruited subgroup of users (n=36).

## Methods

### Patient Process for MS in the Neurology Outpatient Clinic

In Finland, MS is diagnosed solely in the public sector. The neurological outpatient clinic at the Meilahti Hospital covers the publicly organized health care of neurological diseases in Helsinki (0.66 million inhabitants, situation December 31, 2022 [24]). The diagnosis is confirmed with neurological examination, magnetic resonance imaging, and cerebrospinal fluid testing [25].

Follow-up of pwMS is organized as preplanned visits and phone appointments with a neurologist and a nurse. After diagnosis, most patients start immunomodulatory therapy requiring laboratory testing before and during treatment. Usually, a magnetic resonance imaging scan is performed after 6 months of medication initiation followed by a doctor’s appointment. In addition, most patients have an appointment with a nurse to discuss in more detail the practicalities of medications and follow-up investigations. Newly diagnosed patients are invited to a group meeting with professionals giving information on the disease where patients have the chance to answer questions and meet peers. These events are held twice a year. During the disease course, patients will have preplanned visits to their neurologist at 6- to 12-month intervals, with additional visits if needed. In addition, patients will contact a nurse if they have new symptoms or questions between the visits. In 2022, Meilahti outpatient clinic had a care relationship with 1115 pwMS.

### Contents of the DCP

The MS DCP was developed jointly by the staff and patients of the Helsinki University Hospital (HUS) neurology outpatient clinic in 2018-2020. The idea for the DCP started from the common feedback from pwMS that more support is needed after receiving a diagnosis and that there is health care practitioner-dependent variation in the information given about support services. In addition, pwMS found it difficult to get in touch with the outpatient clinic. The DCP aimed to (1) act as a patient education platform for pwMS, providing up-to-date and reliable information on MS, (2) standardize the information and support for all patients, (3) support the adaptation to a chronic illness of patients newly diagnosed with MS, (4) provide easier contact with the outpatient clinic, and (5) enable the timely use of resources for the patients in most need of health care appointments.

Thus, it was developed mainly with the newly diagnosed pwMS in mind. The DCP operates on the MyPath platform in the Digital Health Village, a digital service provided by all 5 University Hospitals in Finland. The Digital Health Village supports the traditional care paths with digital services and consists currently of 33 hubs, 400 DCPs, and 9 online knowledge centers [26]. The use of DCP is voluntary, does not rule out face-to-face contact at the outpatient clinic, and is free of charge for the patients. The service may be used by a mobile device or computer with an internet connection. The DCP is available 24/7 during the whole care relationship at the outpatient clinic.

The DCP contains information about MS, its treatment, and relapses, and provides support for the adaptation to living with a chronic disease, cessation of smoking, starting physical exercise, and coping at work. The adaptation training course of the DCP provides support to the patient after receiving the diagnosis. The DCP also contains questionnaires on symptoms and functional abilities which are encouraged to be filled in before a doctor’s appointment. In addition, the patient has the possibility of sending a message to the health care professional (HCP) at the neurological outpatient clinic, and a video appointment can be launched as well. Patients can store their individual history of MS including time of diagnosis, used medications, social reimbursements, and rehabilitations received on the DCP for their own purposes, for example, used in other contacts with health care. The information content of the DCP is given on videos by professionals and patients sharing their expertise and experiences, on written text and self-assessment questionnaires, and by linking to other reliable websites. The specific contents sections of the DCP are as follows:

1. Welcome to the DCP: information on the DCP and its contents. Both written text and a video (1:06 minutes) with a neurologist introducing the DCP’s different sections and how the DCP works.
2. My MS: a questionnaire about the relevant background information on the DCP user. Questions related to health conditions, ability to work, social benefits such as unemployment benefits, relevant health habits, possible rehabilitation, and how long the special reimbursement for MS medications is valid. This is meant to make the doctor’s appointment smoother. The questionnaire is not standardized. It was developed for the DCP by the staff and patients of Meilahti Hospital.
3. Symptom questionnaire: a questionnaire about neurological symptoms often associated with MS. For all listed symptoms patients report whether they are asymptomatic, if the symptom is an old one, if an old symptom has become stronger, or if it is a completely new symptom. Patients are also asked to estimate how long they can walk, if they have problems with their medication, and what they would like to talk about with their doctor. The purpose of the questionnaire is for patients to fill it out before doctors’ appointments to make the appointments smoother, personalized, and more purposeful. The questionnaire is not standardized. It was developed for the DCP by the staff and patients of Meilahti Hospital.
4. Information on MS: general information on MS. Links to reliable sources of information. A video (3:23 minutes) with two pwMS talking about finding information on the internet about MS, urging to be critical of the found information, what are reliable sources of information, and finding peers on online platforms.
5. Medication: information on medical treatment of MS. Links to reliable sources of information. Information on treatment of relapses with corticosteroid pulse therapy.
6. Adaptation training course: an adaptation training course to support the patient after receiving the diagnosis. The adaptation training course was modified to fill the needs of patients with MS from a template created by a psychologist for all DCPs at HUS and contains information on the psychological process using the following steps:
  Step 1: shock. The first phase of shock and the different ways individuals will react. There are tools for how to cope at this stage and information on where to seek help from an HCP. A video (1:53 minutes) with two pwMS talking about how to accept the diagnosis.
  Step 2: what has happened? The stage where the individual will start to confront the thought of MS. There are rehearsals to support this stage.
  Step 3: how can I cope with MS? The stage where the individual will start to accept the situation. Encouragement is given to start seeking information on MS.
  Step 4: a part of life: the stage where MS becomes a part of life, and the individual’s thoughts are turning to the future. How to take care of one’s resources. A video (1:40 minutes) with two pwMS talking about peer support and different forms of peer support.
7. Support for working life: information on the ability to work, and on how to maintain one’s ability to work. Links to reliable sources of information are provided.
8. Physical exercise and MS: what the importance of physical exercise is in MS. A video (3:13 minutes) of two pwMS talking about physical exercise and how to find the right sport. A link to another DCP which is developed to help people start exercising is provided.
9. Quit smoking: the negative impact of smoking on MS and information on how to obtain support with quitting.
10. Give feedback: questionnaires on patient satisfaction and response to the DCP. Links to technical and content feedback are provided.
11. Send a message: the patient has the possibility of sending a message to the HCP at the neurological outpatient clinic.

### Population and Study Design

All patients under the follow-up for MS in the neurology outpatient clinic at Meilahti Hospital were offered access to the DCP. Data on DCP user behavior were collected on all users of the DCP up to the end of 2022. Data was collected retrospectively and is descriptive in nature.

To evaluate the differences in health care use, a retrospective case-control study was adopted. A case group (n=63) was constructed of the patients who had logged into the DCP at least once and fulfilled the inclusion and exclusion criteria below. A control group (n=112) was constructed of those who had not logged into the DCP but fulfilled the criteria. Data are analyzed with statistical methods.

The inclusion criteria for the health care use part of this study were (1) diagnosed between January 1, 2020, and December 31, 2022, (2) aged ≥18 years, (3) a diagnosis of RRMS, and (4) a recorded Expanded Disability Status Scale (EDSS) score.

The exclusion criteria for this part of this study were (1) a diagnosis of primary-progressive multiple sclerosis (PPMS) and (2) a diagnosis of secondary-progressive MS.

Recently diagnosed pwMS were recruited to a prospective substudy on PROMs from February 1, 2021, to August 30, 2022. The functional impact of MS and patient satisfaction with DCP was assessed, which required filling out surveys. Altogether 118 patients were given access to the MS DCP during the recruitment period, of whom 72 gave initial consent for this study. Of these, 35 were excluded due to a longer time from diagnosis or disease subtype of PPMS, and 1 patient decided to discontinue this study before the end of 12 months. Thus, the final study cohort of PROM consisted of 36 patients. The changes in PROMs are analyzed descriptively.

The inclusion criteria for this part of this study were (1) informed consent, (2) age ≥18 years, (3) ≤3 years from the diagnosis of RRMS at the time of consent, (4) fluent in Finnish language, and (5) experience in using and access to a computer or a smart device.

The exclusion criteria for this part of this study were (1) a diagnosis of PPMS, (2) a diagnosis of secondary-progressive MS, and (3) a severe psychiatric disease (eg, severe depression or a psychotic disease).

It should be noted that while the 3 samples (user behavior, health care use, and PROMs) used in this study are largely overlapping, they are not completely the same. Newly diagnosed patients were emphasized because the principal intended impact of the DCP is focused on newly diagnosed patients.

### Measures

User behavior on the DCP was evaluated by descriptive statistics on the number of users, the number of logins, the timing of logins, and messages sent. These outcomes were chosen as simple metrics of how well the DCP is adopted by pwMS and at what point in their patient journey they use the service.

The health care use between the intervention group and the control group was evaluated using 7 simple quantitative measures: number of physical nurse contacts, number of physical doctors’ contacts, number of remote nurse contacts, number of remote doctors’ contacts, number of other service contacts (including occupational therapy, neuropsychological services, physiotherapy services, rehabilitation services, speech therapy, social worker services, and nutritionist services), number of emergency department (ED) visits, and number of inpatient days. For the first 5, only contacts to the neurological unit were considered as these were assumed to be related to MS. For ED and inpatient days no such filter was included. These measures were selected as the authors believed these can be used to accurately evaluate resource use overall. It should be noted that nonscheduled calls and messages to the MS nurses were not registered in the electronic health record (EHR) as separate contacts and were thus left out of the analysis.

Several factors were considered potential confounders in statistical analyses related to health care use. Previous research has found gender disparities in health care use for pwMS [27]. Age at diagnosis was considered, although previous research has not found a clear association between age at diagnosis and health care use [28]. The EDSS score was considered as a proxy for disabilities, which are known to be associated with higher health care use [29]. Whether or not the pwMS experienced at least one relapse during this study period was considered as a binary variable for disease activity, which is known to be a predictor of health care use [30].

The MSIS-29 [31] was used for measuring the physical and psychological impact of MS during a 1-year follow-up in a prospective substudy cohort. The MSIS-29 self-report questionnaire allows for a detailed evaluation of a patient’s functional health and well-being, encompassing physical limitations, emotional well-being, and overall quality of life. The Finnish version of the MSIS-29 has also been found valid and reliable in previous research [32]. The MSIS-29 score was collected from the patients of DCP at the 0-month, 3-month, 6-month, and 12-month marks. The scores were converted into a scale from 0 to 100 for ease of interpretation. A change of 7-8 points for the physical score [33-36] and a change of 4-6 points for the psychological score [35,36] is considered clinically significant. A higher score indicates higher perceived disability.

The NPS was used to assess the satisfaction of the DCP users at 3 key intervals: 3, 6, and 12 months. NPS is based on a simple question of whether the patient or user would recommend the service to others (rated from 0 to 10), and it has been widely adopted from business to health care [37]. NPS enables the comparison of patient satisfaction between different kinds of services and during service development. NPS is calculated as the percentage of promoters (rated 9 or 10) minus the percentage of detractors (rated 0 to 6). For reference, a previous study on a digital solution for MS care considered an NPS of over 0 “good,” over 20 “favorable,” and over 50 “excellent” [38].

### Data Extraction

The quantitative data on user behavior (number and timing of logins, and number of messages) describing the use of the DCP were extracted from Power BI (Microsoft Corp) reports, and the patient information was gathered from EHRs Uranus (Consultants to Government and Industry Incorporated) and Apotti (Epic Systems Corporation) reporting tools. Patient-reported outcomes (MSIS-29 and NPS) were filled electronically in the DCP platform.

### Statistical Analysis

Statistical analyses were performed with RStudio software (version 2022.12.0+353; Posit Software, PBC). Demographic data were presented in means and SDs as well as medians with minimum-maximum range and IQR. The Mann-Whitney *U* test was used in comparing the means of continuous variables, not requiring an assumption of normality [39]. Similarly, the medians of continuous variables were compared using the nonparametric Mood median test. A *P* value of <.05 was considered statistically significant.

Multivariable linear regression models [40] were used to estimate the effect of the MS DCP on health care use in the presence of confounders. Further, 3 models with different confounder combinations were used to estimate the effect of the MS DCP on support service use. In addition, models with ED visits, inpatient days, and total elective visits were considered.

### Ethical Considerations

This study has been approved by the HUS review board (HUS/2059/2020). The data processing practices followed the European Union Data Protection Directive Rules. This paper conforms with the applicable STROBE (Strengthening the Reporting of Observational Studies in Epidemiology) statement.

## Results

### User Behavior on the MS DCP During 2020-2022

**Figure 1:**
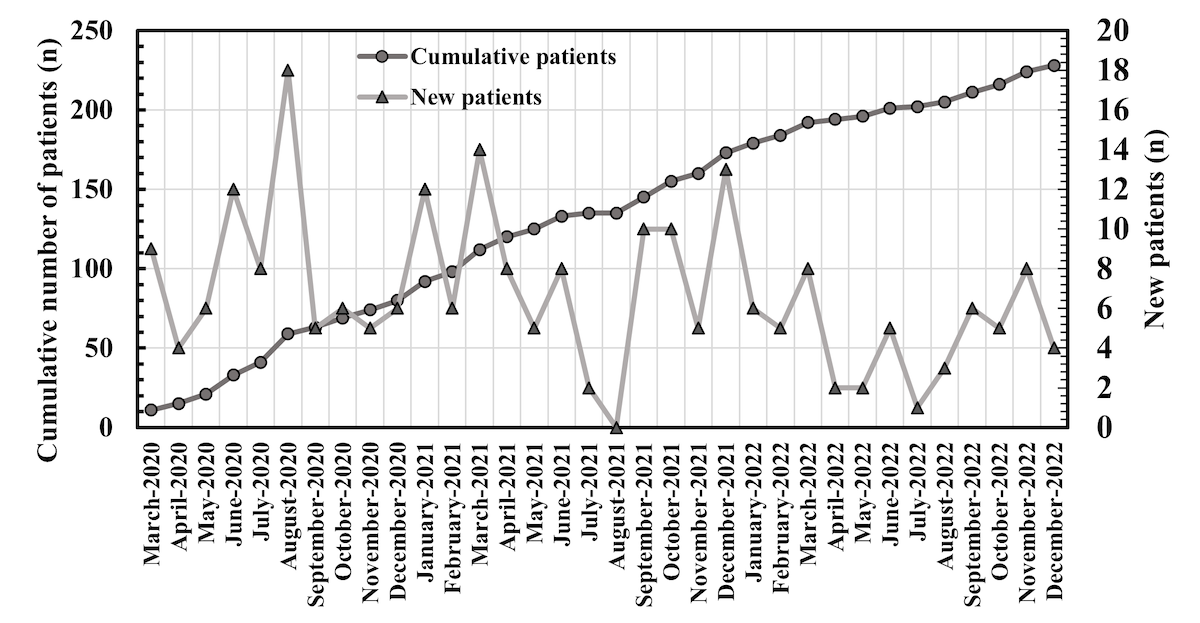
Cumulative number of patients (circle) and number of new patients (triangle) in the Neurology Outpatient Clinic using the DCP shown from March 2020 until December 2022. DCP: digital care pathway.

**Figure 2:**
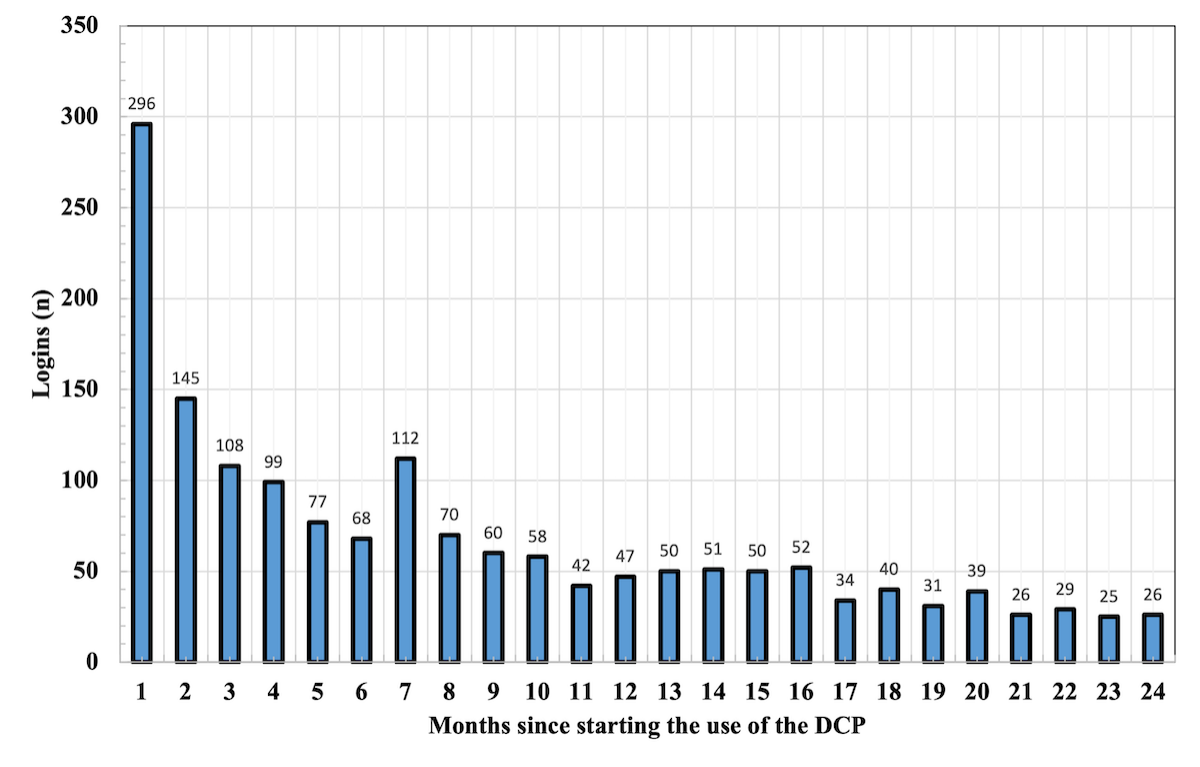
Summative number of logins to the DCP for all pwMS under follow-up in the Neurology Outpatient Clinic. The data are calculated from the time each individual first entered the DCP until 24 months. DCP: digital care pathway; pwMS: people with multiple sclerosis.

The DCP for MS was launched in March 2020 and has since had a steady flow of new users (Figure 1). Overall, as of the end of 2022, a total of 225 patients have had the option to use the service, of which 79.1% (178/225) have logged in to the service. This equals to 16% (178/1115) of all pwMS under follow-up in the Meilahti outpatient clinic. On average, a user of the MS DCP has logged in to the portal 7.4 times. As is evident from Figure 2, most sessions on the DCP take place shortly after the initiation of the access. Over the 2-year observation period starting from the initiation of the service, 18.1% (296/1635) of logins take place during the first month, 72.29% (1182/1635) of logins happen during the first year, and 27.7% (453/1635) during the second year. A minor peak in logins is seen 6 months after the launch of the service, coinciding with a common follow-up visit time. The data on which contents the patients have engaged with in the DCP is not available.

During the observation period, patients using the DCP have sent 1213 messages (average of 6.8 messages/patient) to HCP, usually MS nurses, who have answered the messages roughly in 1 working day. On the other hand, questionnaires on symptoms and functional abilities have been used on average once per patient, even though they were advised to fill it in before every appointment with the doctor.

### Health Care Use

Data on health care use was retrieved from EHRs for a total of 175 patients who fulfilled the inclusion criteria during the observation period. They were separated into an MS DCP user group (n=63) and a control group (n=112). The MS DCP group was made up of the patients who had logged into the DCP at least once. The rest of the patients made up the control group. The groups had similar gender distributions of 73% (46/63) and 70% (79/112) of women, respectively, which well represents the overall female-to-male ratio in the prevalence of MS of roughly 2.35:1 [41]. The mean age was 34.7 (SD 9.83) years in the DCP group and 35.5 (9.76) years in the control group with no significant difference between the two. Similarly, the mean EDSS scores were 1.56 (SD 1.26) and 1.63 (SD 1.19), respectively, with no significant differences. The control group had a higher proportion of pwMS who had relapsed at least once with a total of 13 (N=112, 11.6%) patients, in comparison to only 4 (N=63, 6.4%) in the MS DCP group. This difference, however, was not statistically significant. The characteristics of the MS DCP group and the control group are presented in Table 1.

There were no differences between the MS DCP group and the control group in physical doctor appointments (median 2 visits for both groups), remote doctor appointments (median 8 and 7 contacts, respectively), physical nurse contacts (median 1 visit for both groups), and remote nurse contacts (median 0 visits for both groups; Table 2). Similarly, there was no difference between the groups in emergency care visits (median 0 for both groups) nor inpatient days (median 0 for both groups). A statistically significant difference between the groups was found in other service contacts with the MS DHP group having a median of 1 (minimum-maximum: 0-27) and a mean of 2.57 (SD 4.10) in other service contacts, in contrast to the control group with a median of 0 (minimum-maximum: 0-25) and mean of 0.58 (SD 2.47).

To test for the effects of possible confounders, linear regression models were used (Table 3). The first 3 models tested the association of the DCP and support service use in the presence of confounders. Model 1 considered only the MS DCP, model 2 considered the DCP, gender, and age, and model 3 considered the DCP, gender, age, EDSS score, and relapses. The MS DCP was found to be associated with higher other service use (1.99, 1.95, and 2.05 visits) in all models tested. In addition, linear models were used to confirm no association of the MS DCP with ED visits, inpatient days, and the total number of elective visits (physical or remote, nurses or doctors). No association was found between them.

**Table 1:** Patient characteristics of the health care use study and PROM^a^ cohorts.

Characteristics			MS^b^ DCP^c^ (n=63)		Control (n=112)		Total (n=175)		PROM subgroup (n=36)
**Gender, n (%)**									
	Female	46 (73)		79 (70.5)		125 (71.4)		24 (66.7)	
	Male	17 (27)		33 (29.5)		50 (28.6)		12 (33.3)	
**Age at diagnosis (years)**									
	Mean (SD)	34.7 (9.83)		35.3 (9.76)		35.1 (9.76)		36.2 (9.9)	
	18-24, n (%)	11 (17.5)		10 (8.9)		21 (12.0)		3 (8.33)	
	25-34, n (%)	23 (36.5)		51 (45.5)		74 (42.3)		13 (36.1)	
	35-44, n (%)	18 (28.6)		33 (29.5)		51 (29.1)		14 (38.9)	
	45-54, n (%)	9 (14.3)		11 (9.8)		20 (11.4)		4 (11.1)	
	55 and older, n (%)	2 (3.2)		7 (6.3)		9 (5.1)		2 (5.6)	
**EDSS^d^ score**									
	Mean (SD)	1.56 (1.26)		1.63 (1.19)		1.60 (1.22)		N/A^e^	
	Median (minimum-maximum; IQR)	1.5 (0-5.5; 1)		1.75 (0-5; 1)		1.5 (0-5.5; 1)		N/A	
**Relapse occurred (yes or no)**									
	Total, n (%)	4 (6.4)		13 (11.6)		17 (9.7)		N/A	

^a^PROM: patient-reported outcome measure.

^b^MS: multiple sclerosis.

^c^DCP: digital care pathway

^d^EDSS: expanded disability status scale.

^e^N/A: not applicable.

**Table 2:** Difference in health care use between the MS^a^ DCP^b^ group and the control group.

Resource		MS DCP (n=63)	Control (n=112)	*P* value
**Physical doctor appointments**				
	Mean (SD)	1.68 (0.78)	1.64 (0.90)	.75
	Median (minimum-maximum; IQR)	2 (0-4; 1)	2 (0-4; 1)	.20
**Remote doctor contacts^c^**				
	Mean (SD)	7.60 (3.33)	8.0 (4.55)	.95
	Median (minimum-maximum; IQR)	8 (0-20; 3.5)	7 (0-26; 5)	.05
**Physical nurse appointments**				
	Mean (SD)	1.48 (2.84)	1.16 (2.11)	.90
	Median (minimum-maximum; IQR)	1 (0-13; 1)	1 (0-12; 1)	.29
**Remote nurse contacts**				
	Mean (SD)	0.11 (0.41)	0.10 (0.46)	.67
	Median (minimum-maximum; IQR)	0 (0-2; 0)	0 (0-4; 0)	.66
**Other service contacts^d^**				
	Mean (SD)	2.57 (4.10)	0.58 (2.47)	<.001
	Median (minimum-maximum; IQR)	1 (0-27; 3)	0 (0-25; 0)	<.001
**Emergency department visits**				
	Mean (SD)	0.51 (1.0)	0.69 (2.09)	.88
	Median (minimum-maximum; IQR)	0 (0-4; 1)	0 (0-15; 1)	.92
**Inpatient days**				
	Mean (SD)	0.22 (0.71)	0.38 (1.23)	.95
	Median (minimum-maximum; IQR)	0 (0-4; 0)	0 (0-6; 0)	.69

^a^MS: multiple sclerosis.

^b^DCP: digital care pathway.

^c^Includes asynchronous contacts (ie, administrative-like work, with no direct contact with the patient).

^d^occupational therapy, neuropsychological services, physiotherapy services, rehabilitation services, speech therapy, social worker services, and nutritionist services.

**Table 3:** Regression analysis of the effect of the DCP^a^ use and confounders on health care use (N=175).

Variables	DV^b^: other service use^c^			DV: emergency department visits	DV: inpatient days	DV: all elective^d^
MS^e^ DCP	1.99^f^ (0.50)	1.95^f^ (0.49)	2.05^f^ (0.48)	–0.13 0.28)	–0.12 (0.16)	0.11 (0.81)
Gender: female	N/A^g^	0.39 (0.52)	0.21 (0.51)	0.13 (0.30)	0.18 (0.17)	–0.96 (0.87)
Age at diagnosis (years)	N/A	–0.05^h^ (0.02)	–0.07^i^ (0.02)	–0.03^h^ (0.01)	–0.01 (0.01)	–0.14^f^ (0.04)
EDSS^j^ score	N/A	N/A	0.63^i^ (0.19)	0.13 (0.11)	0.11 (0.07)	0.73^h^ (0.33)
Relapses	N/A	N/A	1.21 (0.79)	1.08^h^ (0.45)	0.95^f^ (0.27)	3.09^h^ (1.33)
Constant	0.58 (0.30)	2.20^h^ (0.98)	1.71 (0.96)	1.19^h^ (0.55)	0.36 (0.33)	15.2^f^ (1.63)
Adjusted *R*^2^	0.080	0.10	0.15	0.03	0.08	0.08

^a^DCP: digital care pathway.

^b^DV: dependent variable.

^c^Occupational therapy, neuropsychological services, physiotherapy services, rehabilitation services, speech therapy, social worker services, and nutritionist services.

^d^All elective: sum of remote and physical nurses’ and doctors’ appointments.

^e^MS: multiple sclerosis.

^f^*P*<.001.

^g^N/A: not applicable.

^h^*P*<.05.

^i^*P*<.01.

^j^EDSS: Expanded Disability Status Scale.

### Patient-Reported Outcomes

PROMs were assessed in a cohort of 36 patients (Table 1), with an initial response rate of 97% (35/36). The MSIS-29 looks for clinically important changes in functional health. A change of 7-8 points for the physical score [33-36] and a change of 4-6 points for the psychological score [35,36] is considered clinically significant. There was a large variability between the MSIS-29 scores reported (Tables 4 and 5). Over the course of this study period, there was no clinically significant change in the median physical MSIS-29 scores, but a clinically significant change was observed for the psychological MSIS-29 scores between the 3-month and 6-month points (an improvement from 22.2 to 16.7). The score, however, rose at the 12-month mark. Of the patients who reported MSIS-29 scores for the 0- and 12-month marks, the change in the median physical score was +3.3, and in the psychological score –3.7. The response rate declined during the year-long study period from 97.2% (35/36) to 47.2% (17/36).

An NPS index was calculated at 3 time points: 3, 6, and 12 months after first login to the DCP (Tables 4 and 5). The NPS at the 3-month mark was 21 indicating a “favorable” reception. At the 6-month mark increased to 30 and at the 12-month mark up to 63 passing the “excellent” limit. The response rate dropped from 80.5% (29/36) down to 44.4% (16/36) at 12 months.

**Table 4:** Patient-reported outcome measures in this study subcohort (N=36).

Measure			Score, median (minimum-maximum; IQR)		Response rate, n (%)
**MSIS-29^a^ physical score**					
	0 months	11.7 (0-78.3; 17.5)		35 (97)	
	3 months	11.7 (0-73.3; 19.2)		31 (86)	
	6 months	10.8 (0-68.3; 20.8)		26 (72)	
	12 months	15.0 (0-86.7; 31.7)		17 (47)	
**MSIS-29 psychological score**					
	0 months	22.2 (7.4-85.2; 31.5)		35 (97)	
	3 months	22.2 (0-63.0; 18.95)		31 (86)	
	6 months	16.7 (3.7-44.4; 17.6)		26 (72)	
	12 months	18.5 (0-59.3; 22.2)		17 (47)	

^a^MSIS-29: Multiple Sclerosis Impact Scale.

**Table 5:** Patient-reported outcome measures in this study subcohort (N=36).

Measure			Score, n		Response rate (%)
**Net promoter score index**					
	3 months	21		29 (81)	
	6 months	30		23 (64)	
	12 months	63		16 (44)	

## Discussion

Here, we described the contents of MS DCP developed in HUS, user behavior on the DCP, differences in health care use between pwMS using the DCP and those who do not, presented results on changes in perceived impact of MS on both physical and psychological functional health of the MS DCP users, and showed patient satisfaction with the service. We show that the DCP has seen a high adoption rate of 79% (178/225), with most logins taking place within the first year after diagnosis (1182/1635, 72.29%). We find a significant increase in other services use and no other differences in the use of services. There was no clinically significant change in the physical MSIS-29 score and while the psychological score oscillated, a clinically significant change was observed between months 3 and 6. Patient satisfaction as measured with NPS was high and improved during this study period (from 21 “favorable” to 63 “excellent”).

The pwMS in this study were young (Table 1), and age is known to be associated with activity in telemedicine services [42]. Thus, patients with RRMS appear to be an exceptionally well-suited patient group to address through digital health care, as the disease is usually diagnosed at an early age [43] and the intensity of the care required is often highest in the beginning when patients need the most support [44]. The impact of a web-based educative and cognitive training program has been demonstrated in a group of patients with MS in the United States and Germany having depression. Both quality of life and psychological health improved, more so with a therapist supporting the training [45]. In this context, the DCP we describe in this study has most content targeted exactly at the early stages of follow-up.

The results also indicate that those using the DCP find their way into available other (supportive and rehabilitative) services significantly more often than those who do not, with on average 2.05 (SD 0.48) more visits during the year after diagnosis than in the control group. This association stayed strong even when accounting for possible confounders: gender, age, EDSS score, and relapses (Table 3). Assuming the cost-effectiveness of rehabilitative and preventative services, in lieu of overtreatment, this could be assumed to be resource-efficient and a positive development both from the perspective of the health care organization and the patient. However, to demonstrate this, a longer follow-up study would be required to show that pwMS using support services either use less health care or have better health outcomes in the future. We found no change in other measures, which aligns well with the fact that the DCP was intended to be a complementary service rather than replacing some part of the regular patient with the MS pathway of the neurological clinic. Finally, no difference in ED or inpatient days was found. All these findings remained when accounting for confounders (Table 3).

Our findings of the DCP being resource-efficient are in line with previous literature on other chronic diseases from Finland [46,47] and elsewhere [48]. In addition, we assume that our analysis underestimates the resource effects of the DCP because, although the health care use data lacked data on most unscheduled remote nurse contacts, unpublished results of analysis in HUS suggest that the nurse time needed for answering 1 patient phone call is 8 times longer than that needed for answering a message. The MS DCP supports messaging as the means of interaction.

A significant improvement in the psychological score was observed between the third and sixth months. Although this later increased, this is an initial indication that there may have been less psychological burden on the patients, which could be attributed to the DCPs educative role or possible time passing and the patient’s coming to terms with the disease. The response rate reduced a lot from 97% (35/36) at 3 months to 47% (17/36) at 6 months. Although this gives uncertainty to interpretation, there is also a possibility that those pwMS who had the biggest improvement dropped out of this study and stopped logging onto the DCP as it had already done its job and supported them with the start of their MS patient journey. Over the 0-month, 3-month, 6-month, and 12-month marks, there were no significant changes [33,34] in the median MSIS-29 physical scores of the patients with MS DCP indicating that there is at least no adverse effect of using the DCP, which was expected.

Patient satisfaction with care is central to the success of any health care delivery model. We used the NPS as a measure of satisfaction with the DCP. Overall feedback was generally positive, indicating a successful adoption of the DCP, with the final NPS index being 63 “excellent.” Again, however, the response rate dropped during this study period from 81% (29/36) down to 44% (16/36), which together with the increasing trend could be an indication that those who were most satisfied with the service kept coming back to it. The MS DCP has several attributes that can be thought of as contributing to patient satisfaction such as patients having access according to their own needs and returning to the information content whenever there is a need for revision. Previous literature has come to similar conclusions [46].

Our study’s primary strength was the comprehensive examination of the DCP’s impact on multiple dimensions—user behavior measures on the DCP, health care use with a case-control design, and PROMs on the impact of MS on functional health, and patient satisfaction. Specifically, we present data on all users of the DCP and pwMS diagnosed within a defined period as our sample and follow all these pwMS over a period of 1 year. In addition, the PROMs were collected longitudinally at several time points which further strengthens our study. Considering these aspects, this study provided a well-rounded view of the MS DCP. However, there are some significant limitations. First, the cohorts studied in the different parts of this study do not completely overlap and have somewhat different inclusion and exclusion criteria limiting the possibilities of what can be said by combining insights from the groups. Second, it should be noted that although we found a very strong connection between the MS DCP and the use of other services, it is possible that some mediating variable outside of our analysis (ie, the probability of an individual seeking support) could be affecting the results. This fact also introduces some selection bias into our cohort. Third, the section of this study dealing with PROMs had a large reduction in response rates over this study period. While this can be partially explained by a natural attrition rate in longitudinal studies, the sharp decrease in responses may have introduced bias into the results, with those reporting the highest satisfaction levels being more likely to continue engagement with the DCP.

Future studies should investigate whether there are specific patient subgroups, for example, younger patients or more digitally fluent patients that are more likely to engage with and benefit from the MS DCP. Another area for future research is to explore how the cost-effectiveness of the DCP compares to traditional care models in different health care settings, such as other DCP studies from the Finnish context [46].

In conclusion, digital service is a resource-wise way of importing information about MS and support in the early adaptation phase of the disease to pwMS. Our results warrant further research into the long-term effects of DCPs as complementary solutions to existing pathways in MS, and other chronic conditions as well.

## Abbreviations

DCP: digital care pathway
ED: emergency department
EDSS: Expanded Disability Status Scale
EHR: electronic health record
HCP: health care professional
HUS: Helsinki University Hospital
MS: multiple sclerosis
MSIS-29: Multiple Sclerosis Impact Scale
NPS: net promoter score
PPMS: primary-progressive multiple sclerosis
PROM: patient-reported outcome measure
pwMS: people with multiple sclerosis
RRMS: relapsing-remitting multiple sclerosis
STROBE: Strengthening the Reporting of Observational Studies in Epidemiology
